# Land Use for Edible Protein of Animal Origin—A Review

**DOI:** 10.3390/ani7030025

**Published:** 2017-03-18

**Authors:** Gerhard Flachowsky, Ulrich Meyer, Karl-Heinz Südekum

**Affiliations:** 1 Institute of Animal Nutrition, Friedrich-Loeffler-Institut (FLI), Federal Research Institute for Animal Health, 38116 Braunschweig, Germany; gerhard.flachowsky@fli.de; 2 Institute of Animal Science, University of Bonn, 53115 Bonn, Germany; ksue@itw.uni-bonn.de

**Keywords:** food security, human-edible protein, land use, arable land, grassland, plant yield, animal yield, co-products

## Abstract

**Simple Summary:**

The growing world population has led to a higher demand for more and better quality food. In the future, there will be increasingly strong competition for arable land and other non-renewable resources. Proteins of animal origin are very valuable sources of essential nutrients, but their production consumes resources and causes emissions. The aim of this study was to calculate exemplarily the land use for production of edible animal protein from different animal species and categories in consideration of important influencing factors. Large differences were found with the highest amounts per kilogram of body weight produced by broiler chickens and the lowest yields in edible protein and the highest land need observed for beef cattle.

**Abstract:**

The present period is characterized by a growing world population and a higher demand for more and better quality food, as well as other products for an improved standard of living. In the future, there will be increasingly strong competition for arable land and non-renewable resources such as fossil carbon-sources, water, and some minerals, as well as between food, feed, fuel, fiber, flowers, and fun (6 F’s). Proteins of animal origin like milk, meat, fish, eggs and, probably, insects are very valuable sources of essential amino acids, minerals and vitamins, but their production consumes some non-renewable resources including arable land and causes considerable emissions. Therefore, this study´s objective was to calculate some examples of the land use (arable land and grassland) for production of edible animal protein taking into consideration important animal species/categories, levels of plant and animal yields, the latter estimated with and without co-products from agriculture, and the food/biofuel industry in animal feeding. There are large differences between animal species/categories and their potential to produce edible protein depending on many influencing variables. The highest amounts per kilogram body weight are produced by growing broiler chicken followed by laying hens and dairy cows; the lowest yields in edible protein and the highest land need were observed for beef cattle. This review clearly indicates that the production of food of animal origin is a very complex process, and selective considerations, i.e., focusing on single factors, do not provide an assessment that reflects the complexity of the subject.

## 1. Introduction

Food security is currently a pivotal phrase. Food security means meeting the demand of a growing world population for food of plant and animal origin. The FAO (Food and Agriculture Organization of the United Nations) [1] defined food security as “access to sufficient, safe, nutritious food to maintain a healthy and active life”. This, in turn, is associated with a growing demand for limited natural resources such as land area, fuel, water, and minerals, and with elevated emissions of gases including greenhouse gas (GHG) potential such as carbon dioxide (CO_2_), methane (CH_4_), nitrous oxide (N_2_O), and other substances (e.g., nitrogen, phosphorus, trace elements). 

At the end of October 2011, the seven billionth person was born. Sustainability in feed and food production is a key challenge for agriculture as summarized recently in articles and monographs (e.g., [2,3,4,5,6]). In the future, there will be strong competition for arable land and non-renewable resources such as fossil carbon-sources, water [7,8,9], and some minerals such as phosphorus [10,11]. There will also be competition for land use between feed, food, fuel, fiber, flowers, and fun (6 F’s concept; [12]), as well as between areas for settlements and natural protected areas. 

The balance between Planet (global resources and emissions), People (population all over the world with adequate nutrition and social conditions), and Profit (money-making) in the so-called 3P-concept [7,13] is an important prerequisite for sustainable life and development on the earth. Profit should not be the single objective of production. We have to find a balance between a careful and sustainable use of limited resources on the one hand and low emissions with minimal local and global consequences for later generations on the other hand.

According to the FAO [14,15], the human population will increase globally from above seven billion currently to more than nine billion people in 2050, but the increase in the output of food of animal origin is estimated to be about 70% [16]. Therefore, some authors propose a redefinition and a rethinking of agricultural yield, and agriculture in general, from tons per hectare to people per hectare [17,18] and increasingly demand sustainable animal agriculture [19]. The energy and protein conversion efficiency from feed into food of animal origin is low and may range from 3% (energy—beef) up to 40% (energy—dairy; protein—chicken for fattening) [18,20]. In some countries (e.g., USA) between 67% (energy) and 80% (protein) of the crops are used as animal feed [18]. Feedstuffs such as grass and other roughage which cannot directly be consumed by humans, co-products of agriculture and of the food and biofuel industry may also contribute to a more efficient production of food of animal origin. Especially with regard to ruminants, less potential human food should be fed to animals in order to improve the proportion of protein output by animals relative to protein input [21]. 

These developments and complex connections present the following question: “Is there any need for food of animal origin?” As some population groups (e.g., vegans) demonstrate, there is no essential need for food of animal origin, but the consumption of meat, fish, milk, and eggs may contribute significantly to meeting the human requirements of amino acids [22,23,24] as well as some important trace nutrients (such as Ca, P, Zn, Fe, I, Se, and vitamins A, D, E, B_12_), especially for children and juveniles as well as pregnant and lactating women [25,26]. Human nutritionists [27,28] have recommended that about one third of the daily protein requirement (0.66–1g per kg body mass [22,29]) should originate from protein of animal origin. Consequently, about 20 g of the daily intake of about 60 g protein [30] should be of animal origin, which is lower than the present average consumption throughout the world. Presently, there is an average consumption of animal protein (without fish and insects) of about 24 g per capita and day, ranging from 1.7 (Burundi) to 69.0 g (USA; Table 1). It is difficult to assess the protein intake from fish. Avadi and Freon [31] estimate that half of the world´s population consumes at least 15% of their animal protein from aquaculture.

Other reasons for consumption of food of animal origin are the high bioavailability of most nutrients and their considerable enjoyment value. Such food is also considered to be an indicator of standard of living in many regions of the world, and it is also determined by taste, odor, and texture, as well as by geographic area, culture, ethics, and wealth. Further reasons for the higher demand of food of animal origin in some countries are the increased income of the population [32] and the imitation of the so-called “Western style of life”. Many developing countries continue to consume more animal products than they produce. Therefore, they will continue to drive the world demand for all agricultural products, including food of animal origin [33,34]. Wu et al. [34] estimated that with exponential growth of the global population and marked rises in meat consumption, demands for animal-source protein will increase by 72% between 2013 and 2050. Greater amounts of food of animal origin require greater plant yields and/or more area for feed production, more animals and/or higher animal yields, and an increase in agricultural trade.

On the other hand, changing the eating patterns [35] and eating fewer or no livestock products, especially meat, is a possible solution to reduce the environmental impact of animal agriculture [36,37,38] and to reduce the per capita land requirements [39,40].

Apart from resource needs, feed and food production causes emissions with a certain greenhouse gas (GHG) potential, such as carbon dioxide from fossil fuel (greenhouse gas factor (GHF) = 1), methane (GHF about 23) from enteric fermentation, especially in ruminants, and from excrement management, as well as nitrogen compounds (NH_3_ and N_2_O, GHF about 300; [41]) from protein metabolism in the animals [19,24,42,43,44]. Apart from a lower input of limited resources along the food chain, a low output of GHG, summarized as carbon footprints (CF) and expressed as carbon dioxide equivalents (CO_2_-eq), and minerals such as phosphorus and some trace elements during feed and food production are very important aims of sustainable agriculture. Presently, about 14.5% of total human-induced global emissions, estimated at 7.1 Gt CO_2_-eq annually, come from the global production of food of animal origin [45]. Therefore, as underlined by some authors [43,46,47], more attention should be given to optimizing animal breeding [19,48] and animal health, reducing animal losses, and developing low-emission diets using life cycle assessments (LCA) for all societies [49,50].

**Figure 1:**
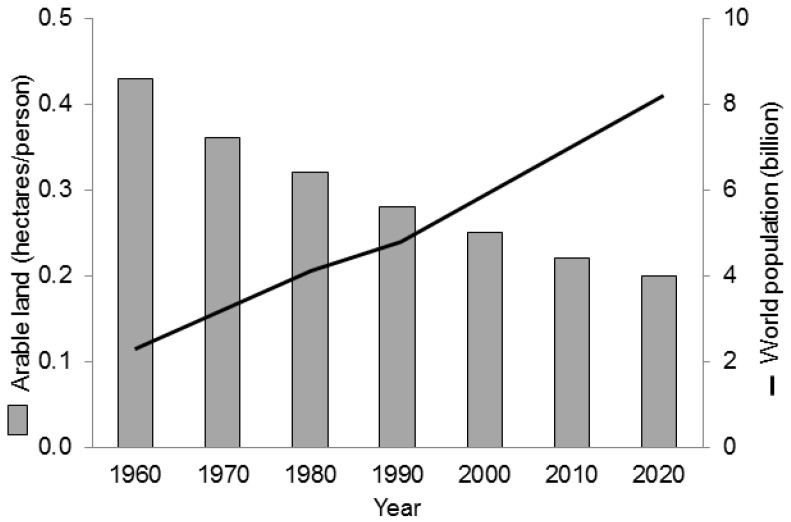
Development of world population compared to arable land per inhabitant between 1960 and 2020 [52].

Land, especially arable land, is one of the most important limiting factors [51]. Only a small portion of the approximately 13.4 billion ha global surface is available as arable land (about 1.5 billion ha, or about 12% of the world’s land area [24,52]). This area could be extended to a degree (by about 120 million ha [24,52]), but some areas cannot be used because of limited water resources, forests, urban settlements, environmental protection, deserts, mountains, and other factors. As a result of the finite area of arable land and the increase in population, the area of arable land available per person decreased from about 0.45 ha (1960) to about 0.25 ha (2010) and will further decrease to below 0.20 ha per person after 2020 (Figure 1).

This situation and the increasing use of land for biofuel production [53], organic farming, settlements, natural protected areas, and other purposes has consequences for feed and food production. Because of the high need for limited resources, attention has been paid to the space needed for animal production. Some authors have considered the land use, also described as land or area footprint, for food of animal origin [38,39,54,55,56]. These authors proposed to distinguish between areas (mostly arable land) that can also be used for purposes other than for feed production (6 F-concept: food, feed, fuel, fiber, flowers, and fun) and typical feed areas (grassland or perennial crops). Reynolds et al. [26] analyzed the importance of animals in agricultural sustainability and food security. Grassland and co-products of agriculture, food, and the biofuel industry do not need arable land and, thus, do not contribute to food-feed competition between animals and humans. The human-edible feed conversion efficiency (heFCE) [57,58] is defined as human-edible output via the animal products (e.g., edible protein) divided by the potentially human edible input via feedstuffs and may contribute to the calculation the feed need without competition to human nutrition. Van Zanten et al. [59] proposed the calculation of land use efficiency of livestock systems to address the contribution of livestock to the global food supply. 

There is no reason to believe that the public interest in the use of limited resources and the high emissions (i.e., CF) from the production of food of animal origin will diminish in the near future because of the worldwide increase in land need and emissions. Therefore, the objectives of this paper are (1) to make some model calculations for the land use and to propose land footprints (LF) for production of food of animal origin, measured per product unit and/or as edible protein and (2) to compare the data with selected references. Furthermore, some calculations to compare human-edible protein output via food of animal origin with edible protein input are done.

Such data could be considered as a starting point for calculations of land use per inhabitant and year (or lifespan), depending on, for example, the amount of protein consumption, protein sources, and levels of plant and animal yields. Average values for plant and animal yields were used and demonstrated the model character of present calculations. In the future, more details should be considered to help determine the possible or necessary intensity level for feed and animal production in order to have an available amount of food of animal origin for human beings. Alternatives to the production of food of animal origin with minimal use of arable land, and with consideration of grassland and co-products from agriculture and the food and fuel industries, should also be considered.

The structure of this article is a combination of a review and author-based calculations, for which references were selected based on presumed particular relevance in the context of the issues discussed in this study A thorough review would have had the major message that previous attempts to estimate land use for edible protein of animal origin had used very diverse variables and factors—making sound conclusions impossible. For the same reason, a meta-analysis using an appropriate statistical model could not be performed because different authors used different definitions for the same term, and published data did not allow converting data to a common denominator. Without this, however, a meta-analysis would have been subject to erroneous results.

## 2. Materials and Methods 

### 2.1. Estimation of Human-Edible Fractions of Feeds

Food-producing animals, especially non-ruminants, consume plant protein which is also edible by humans. Therefore, the proportion between edible protein input by animals and the output as food of animal origin are questioned (e.g., [18,36,39]). The human edible feed conversion efficiency (heFCE) can be calculated on the base of human-edible output in the form of animal products divided by potential human-edible input [57,58]. Such calculations can be done for edible protein (heP; see Table 2) of feed, but also for edible energy (heE). The data shown in Table 2 are used to calculate the heFCE. Wilkinson [57] proposed values for the calculation of heFCE of rations for human-edible energy (heE) and human-edible protein (heP): 80% for cereal grains, pulses, and soybean co-products; 20% for wheat bran, sunflower, and rapeseed co-products and no heE/P (i.e., 0%) for grass, silages, and all other co-products. Some values applied by Wilkinson [57] are disputable (e.g., for maize silage), because the feeds are produced on arable land, which could alternatively be used for cultivation of plants with high human-edible fractions (hef).

Some authors (e.g., [21,22,52]) consider the quality and digestibility of potentially human-edible inputs (plant protein) to describe the nutritional value of proteins as the protein quality ratio (PQR). Such calculations are based on the limiting amino acid in dietary protein, divided by the same amino acid of the reference protein [61] or further derived equations [24,52]. This approach adds further quality but also complexity to the estimation of heP which can make comparisons across sources difficult.

### 2.2. Edible Fraction and Protein Content of Animal Products

The production of protein of animal origin is one of the most important goals of animal husbandry [5,38,55]. Edible protein can be used to compare the efficiency of animal production (e.g., milk, meat, eggs) and to assess the emissions per product [62].

Quantification of protein yield varies depending on some influencing factors. For example, milk and eggs are clearly defined as food of animal origin and the yield can be measured (and expressed as kg or L per animal or per day), and, therefore, it is relatively easy to use the yield of lactating and laying animals for further calculations. The edible fraction of milk and eggs is almost 100% of the yield. Only minor fractions are not consumed by humans (e.g., colostrum, milk samples at the beginning of milking, egg membranes, and shells), which are not considered in further calculations (Table 3).

It is much more difficult to quantify and characterize the yield from the animal body after slaughtering and processing. The following endpoints can be measured in the case of animals for meat production:

- Weight gain of the animal (per day or per growing period) during the whole life span
- Weight gain of the animal without gastro-intestinal tract contents
- Empty body mass (or carcass weight; meat and bones; warm or cold)
- Meat (empty body mass minus bones)
- Edible fraction (meat plus edible organs and tissues)
- Edible protein (edible fractions of the carcass multiplied by their specific protein content)

Mostly, the term “meat” is used, but sometimes what is exactly meant by this term is not clearly described (meat with or without bones). Peters et al. [63] introduced the term “hot standard carcass weight” (HSCW) as the weight at the exit gate of the meat processing plant. The FAO [24] defines meat from animals as fresh, chilled, or frozen meat with bones. The FAO data on meat are given in terms of dressed carcass weight excluding offals and slaughter fats. The HSCW varies between 50% and 62% of the live weight of cattle before slaughter, but it may vary between 50% in the case of sheep up to 80% for turkeys [38,42,63,64]. Nijdam et al. [38] used the following killing-out percentages (carcass weight as a percent of live weight): 53% for beef, 75% for pork, 46% for mutton, 70% for poultry, and 40% for fish. The edible meat yield (retail meat of carcass), given by the same authors, is: 70% for beef, 75% for pork and mutton, 80% for poultry, and 100% for fish.

When it comes to the definition of “edible” fractions, large differences exist between countries, and also between population groups within one country. Therefore, it is difficult to compare results from various authors and to find out the actual protein yields. Regardless of the method used for calculations of edible fractions, authors should always clearly describe how values were used and derived to allow understanding and interpretation of results [63]. Another important factor for a reliable calculation of protein yields of food of animal origin is the protein content of edible fractions as shown in Table 3.

The results of specific studies (Table 3) as well as values from food tables [70,74] are, overall, in agreement regarding the ranges of protein content in animal-based food: milk (between 30.8 and 37.0 g/kg), beef (170–227 g/kg), pork (129–240 g/kg), poultry meat (182–242 g/kg), and eggs (110–130 g/kg). Much lower values for beef, pork, and poultry were used by [71] to calculate water footprints for food of animal origin, resulting in very high values for some foods of animal origin (e.g., [71,75]). The protein yield in the edible fractions of milk, meat, and eggs was calculated based on our previous assumptions and data [65] (Table 3).

### 2.3. Animal Performances/Yields

Apart from the edible fractions and the protein content of edible fractions, the protein yield per animal and day is also influenced by animal species and categories and by animal performance. Table 4 summarizes data about the animal species/categories and the performance of animals regarding their expected yield of edible protein. This paper will consider edible protein of animal origin as the main objective of animal husbandry. Furthermore, it is also easier to compare animal yields of various forms of animal production based on animal protein yield [38,55,62].

The highest protein yields per kg body mass are from intensively produced growing and laying poultry, followed by lactating ruminants and pigs. Growing ruminants (e.g., beef cattle, suckler calves) show the lowest protein yield per kg body mass and day (Table 4). Table 4 only shows data of animals in the production phase. Yet, animal production is more complex and for more detailed and reliable calculations, the breeding/reproduction phase should also be considered. If these phases are taken into account, yields are lower and it also becomes evident that impaired reproduction or fertility—often caused by poor nutrition and (or) diseases—is a major obstacle to more efficient resource use by food-producing animals.

### 2.4. Plant Yields on Grassland (Perennial Crops) and on Arable Land

Apart from animal performance, information on plant yields should also be available for land use calculations. The need for arable land is influenced by various factors such as

- Type of forage and crops from arable land and their yields
- Losses during harvest, preservation, and storage
- Consideration of co-products of agriculture, food, and biofuel industries (such as straw, solvent-extracted oilseed meals, dried distiller’s grains with solubles (DDGS) in animal nutrition)
- Animal species and categories, number of animals, and animal yields
- Diet composition; forage-to-concentrate ratio

The easiest way to incorporate plant yields is to calculate the land use on the basis of dry matter (DM) yields, but a more specific characterization of feed (e.g., consideration of various plant species, digestibility, protein yield, or energy yield expressed as digestible, metabolizable, or net energy) would be helpful for a more accurate assessment. Energy and nutrient requirements for all food-producing animals including horses across a wide range of performance levels can be found in documents from the National Research Council of the USA (e.g., for dairy cattle: [76]) or in the recommendations of the Society of Nutrition Physiology in Germany [66,67,68,69,77].

Three intensity levels (A—low, B—medium, and C—high) for plant yields on grassland (perennial crops = roughage) and for arable land (cultivated crops = concentrate) were assumed for further model calculations (Table 5). Low, medium, and high yield levels were considered. The assumed plant yields (Table 6) were selected to show the influence of yield on the land needed per unit of animal product. Higher plant yields are an important prerequisite for solving global nutrition problems [78]. Another point especially significant for ruminants is the digestibility or the feed value of roughage, especially if grown under tropical conditions. Such climatic conditions are expected to result in forage of low nutritive value and a high proportion of protective structures containing cellulose, hemicelluloses, lignin, cutin, and other structural substances such as silica [79]. Tropical forage, therefore, often has a low nutritive value and a high proportion of protective structures to protect the plant against predation, i.e., herbivory. Additional factors, e.g., long warm nights promoting respiration, warmer growth temperatures increasing lignification, and specific types of grasses in the tropics (mostly C4-plants) are also responsible for the lower nutritive value of tropical plants. As an example, the average digestibility of the dry matter of grass/legume mixtures as given by the FAO [24] is 75% for Europe and 64% for Africa, and for conserved grass/legume mixtures, the values are 71% for Europe and 54% for Africa. More country- and feed-specific values are available in specific feed value tables (e.g., [80,81]).

In tropical areas, free ranging animals, mainly ruminants (e.g., cattle, goats, sheep, and deer), consume grassland. They are able to select feed according to their preference [82] and may substantially contribute to supplying food of animal origin under such conditions. No limited resources, such as arable land and fuel, nor extra water supply, are needed for these animals. Future studies should consider the plant yields (Table 6), the nutritive value of grassland and perennial crops, the methods for increasing feed value by intensified plant breeding or the improvement in post-harvest methods before and during storage, and the assessment of consumption of such feeds by ruminants. Lower plants yields and variation in digestibility and feed intake should also be analyzed as they are additional influencing factors on animal yields under tropical conditions.

In the present paper, further calculations were made taking into consideration the yields for forage and concentrates (Table 6) grown under the same yield levels; however, in reality, it is also possible to harvest forage with yield level B and concentrates with yield level C, or to import feeds from regions with lower or higher yields. The significance of co-products for animal feeding is described in Section 2.5.

Ruminants generally do not require grains or other concentrates because the microbial population in the rumen is capable of digesting plant fiber and, after fermentation of the released sugars, delivers energy to the ruminant animal. Ruminants are, therefore, able to produce edible protein of animal origin (milk and meat) from permanent meadows and pastures. They are enabling a net output of human edible protein and may contribute to meeting the human needs for food of animal origin (e.g., [83,84,85]). Kratli et al. [86] pointed out that pastoralists are more efficient at producing food per unit area of dryland than other forms of agricultural land use under the same conditions. Pastoralist systems are also efficient users of resources such as manure [87]. Because of the high fiber content of forage from grassland and the low animal yields, methane emissions and the CF per kg of edible protein may be higher under such extensive conditions [24,88] and GHG mitigation measurements are, therefore, very important [43,44,45,89]. On the other hand, it should be considered that permanent grassland has a quantitatively significant potential for soil carbon sequestration [90,91].

### 2.5. Significance of Co-Products

Apart from roughages and grains, co-products from agriculture (e.g., [60,92,93]), food production [60,94], and the biofuel-industry [95] are commonly used as animal feeds. Co-products are by-products of main processes such as grain production (e.g., straw, stalks, husks) and processing of raw products in the food industry (e.g., solvent-extracted oilseed meals from the vegetable oil industry, bran from cereal grain processing, beet pulp or bagasse from the sugar industry, and animal co-products from milk, fish, or meat processing) or from the biofuel industry (e.g., DDGS, rape seed cake and meal, as well as cakes and meals from other oilseeds). According to the FAO [24], between 10% and 50% of the estimated concentrate feed comes from co-products in various global regions [96]. In some countries, up to 100% of concentrate may be co-products.

Co-products are used in various amounts and proportions in animal diets. Cereal straws and other co-products rich in plant cell-walls are mostly characterized by a low digestibility and are, thus, poor in energy and protein delivery. They are fed to ruminants with low animal yields or just to meet their maintenance requirements. For high yielding ruminants, they can only be considered as a source of fiber. Normally, they are not used in the feeding of non-ruminants.

In co-products from the food and fuel industry, the concentration of those nutrients not used for processing is two to three times higher than that found in the original product (e.g., protein in the case of DDGS). They can be used as valuable sources of protein, minerals, and other nutrients depending on the source material and the chemical or physical processing, without causing any LF. In the future, more grain will be used for food and fuel and more co-products could be available for animal nutrition [21,85,97] or other purposes. Additional details about the nutritive value and utilization of co-products from the biofuel industry in animal nutrition were recently compiled by the FAO [95].

Co-products of agriculture and of the food/fuel industry can be used to replace concentrates and forage from grassland. Based on plant yield level B (Table 5), we assumed for model calculations that agricultural co-products (e.g., cereal straw) may replace 10%, 20%, or 30% of forage from grassland or other sources in all diets, and co-products from the food or biofuel industry may replace 15%, 30%, or 45% of concentrate without any land use in the model calculations of all animal diets.

### 2.6. Animal Feeding

The roughage-to-concentrate ratio is of special importance in ruminant nutrition (Table 4). Ruminants need a certain portion of forage (i.e., structural fiber) for overall health and to sustain normal rumen fermentation processes [98]. The concentrate portion in ruminant diets increases with higher animal yields (Table 4) because of higher energy and nutrient requirements of such animals and limited feed intake capacity [68,76], but a certain level of forage is also required to keep animals healthy. Higher ruminant yields require more concentrates and more arable land; however, the land requirements can be decreased by the strategic and intensified use of co-products. Special attention must be paid to feeding of potential human edible feeds to ruminants, such as cereal grains and pulses [18,99]. Typically, non-ruminants are fed with concentrates or co-products from the food or biofuel industries (Table 4). Animals with lower yields or mature animals (e.g., pregnant, non-lactating sows) may also consume certain amounts of forage in their diets. However, it is nearly impossible to include forage in the diets of higher performing non-ruminant animals without drastically compromising performance [39].

## 3. Results and Discussion

### 3.1. Animal Species and Protein Yields

Table 6 shows the calculated results of yields from perennial crops, arable land (yield levels A, B, and C; Table 5), and animals (Table 4) based on the animal species/categories, whereas Table 4 shows the forage-to-concentrate ratios. Co-products from the food and biofuel industry were not considered as feeds in the calculations of Table 6. Animal species, animal yields, and plant yields were the most important influencing factors on land use.

Lower plant yields (levels A and B) require more area per unit of edible protein, especially in the case of perennial crops. Higher ruminant yields increase the arable land use because of the higher energy density in the diets, but decrease the need for roughage/grassland. Beef cattle produce lower protein amounts (Table 4) and need much more land per unit of edible protein than dairy cows (Table 6).

Only small amounts of forage were used for non-ruminants with lower yields. Higher animal yields from non-ruminants result in a decreased need of arable land per kg of edible protein (Table 6). Non-ruminants need a much greater area of arable land compared to that of ruminants because of the higher concentrate portion in their diets (Table 4). More detailed calculations, which would have gone beyond the scope of this study, are necessary to consider growing and reproductive periods for cows, sows, and laying animals as well as animal losses.

In the following paragraph, our data is compared with selected references. De Vries and de Boer [55] reviewed 16 LCA studies and found—in agreement with the present calculations (Table 6)—that the production of 1 kg of beef or 1 kg of edible beef protein required the most land and had the highest global warming potential followed by the production of 1 kg of pork, chicken, eggs, and milk. There was a similar ranking on the basis of edible protein (beef > pork > chicken > milk > eggs; Table 7). De Vries and de Boer [55] did not distinguish between grassland and arable land, however, this is important to compare total land use with arable land use of ruminants and non-ruminants.

Nguyen et al. [100] calculated the land use of beef produced in different systems and expressed land use per kg of slaughter weight at the farm gate. One kilogram of slaughter weight is approximately equal to about 100 g of edible protein (Table 4 and Table 6). The total land use varied, depending on the production system, between 16.5 and 42.9 m^2^/year (between 0 and 36.9 m^2^ of grassland and between 6.0 and 16.5 m^2^ of cropland). The values per kg of edible protein varied between 165 and 430 m^2^ and are similar to values in Table 7. In agreement with data from Table 6, the largest area of arable land (cereal grains, soybean) was needed for the highest weight gain, whereas more extensive feeding systems resulted in lower weight gains, longer feeding periods, and more grassland use.

### 3.2. Grassland and Arable Land

Arable land is needed for food production, industrial raw materials, or the other F´s, like fuel, fiber, flowers, and fun [12]. Therefore, animal nutrition will be forced to use human-inedible biomass from grassland or co-products from the food and fuel industries to a much greater extent.

On a global scale, grassland could be an important potential source for ruminant nutrition. Sustainable intensification of ruminant farming requires a development of grassland-based forage production [84]. Sustainable grassland management includes avoiding overgrazing and a strategic use of manure to maintain or improve productivity of the swards.

Nijdam et al. [38] analyzed numerous LCA and considered both total land use and the portion which is grassland (Table 8). The calculated values for the land use (m^2^) per product and per kg of protein show a large range and cover all the data shown in Table 6, Table 7 and Table 9. Apart from the total land use, the authors also determined that the grassland portion produced 1 kg of products or 1 kg of protein by ruminants per m^2^. The results demonstrate that a better description of conditions used for calculations (such as plant and animal yields, use of grassland and arable land, as well as co-products in ruminant feeding and slaughtering and protein yields of assessed food of animal origin) are necessary to allow for the comparison of the results of various studies and to draw more reliable conclusions.

### 3.3. Co-Products

As mentioned earlier, the feed sources may remarkably influence the calculation of the land use per kg of product or edible protein. There has been a long discussion about the land area considered for co-products when used in animal nutrition. Some authors consider a part of the area (20%–50%) used for growing the main product (e.g., cereals, oilseeds) as an area for the co-product. However, in other studies, land use was not considered for growing co-products at all [21,58,101,102]. In accordance with this concept, co-products may replace forage and/or concentrates in the diets without any additional LF as shown in Table 6 in comparison to Table 9.

Calculations in Table 9 show examples when up to 30% of grassland areas and up to 45% of arable land areas are replaced by straw or other low quality roughages and by co-products from the food and fuel industries, respectively. Such conditions should be considered if the land uses of various protein sources are compared. Extensive examples for the accepted uses of co-products in farm animal diets were recently summarized in the literature [58,95]. Ertl et al. [58] demonstrated that wheat bran and sugar beet pulp as sole concentrates for dairy cows supported a daily milk production of more than 20 kg per cow under Austrian conditions.

The results of field studies (Table 7 and Table 8) and our model calculations (Table 6 and Table 9, Table 10 and Table 11) clearly show that the study or calculation conditions should be carefully described to allow appropriate understanding and interpretation of data.

### 3.4. Human Edible Protein

The human edible protein yield was calculated by dividing the protein output per edible animal product by the edible protein intake and denoted protein score. Values >1 indicate a net yield in human edible protein, whereas values <1 demonstrate a protein loss via animals. Table 10 shows the effects of milk yield and required concentrate amounts on the human edible protein input and the output per cow as well as the protein score (as defined above; [57,58]). The higher the milk yield and the higher the protein intake from edible concentrate, the lower the protein score. In agreement with the calculated data (Table 10), Ertl et al. [58] calculated protein scores between 1.40 and 1.87 for milk for the potentially human-edible plant protein for 30 Austrian dairy farms with average daily milk yields between 20 and 25 kg.

Table 11 evaluates protein sources for the production of food of animal origin. In the first case, no co-products are used in the diets; in the second case, 50% of concentrate are consumed as co-products, mainly wheat bran, and sugar beet pulp. The data show that in addition to the lower need of arable land (see Table 9), the protein score for all protein sources is higher if co-products are used in animal feeding. Except milk, the protein scores for all food of animal origin are <1 without co-products as part of concentrate (Table 11).

## 4. Conclusions

Arable land use per unit product or protein of animal origin depends mainly on animal species and categories, animal production system, plant and animal yields, use of grassland, and the portion of co-products in animal rations. The results presented here show that a clear and precise description of study conditions and/or the basis of calculations is an inevitable prerequisite to allow for a fair comparison of results concerning land use. More complex calculations, considering characteristics of efficient use of limited resources and the reduction of emissions, seem to be helpful to find and specify optima in the production of food of animal origin. Pertinent references are mentioned for further details. The following aspects and measurements should be considered in future calculations [103]:

- Use of arable land (i.e., competition between various users [2,12])
- Comparison of output of human edible protein with input via feeding (should be >1 [57,58])
- Efficient use of water for feed and animal production [9,75]
- Minimization of the use of fuel and other limited natural resources in the food chain [13,14]
- Utilization of permanent grassland and co-products from agriculture and industry [21,60]
- Reduction of greenhouse gas emissions per product or per kg of edible protein and along the whole food chain [41,42,43,44,45]
- Plant and animal breeding as the starting points of the human food chain [48,78]
- Evaluating the potential of edible insects as protein sources for food and feed [49,104]
- Calculation of land use per inhabitant considering eating patterns of the population [17,35]
- Reduction of food wastage (presently equal to about 1.4 billion ha land or 30% of the world´s agricultural area [105,106]

The production of food of animal origin is a very complex process, and selective considerations, i.e., focusing on single factors, do not provide an assessment that reflects the complexity of the subject. Cooperation of animal scientists (e.g., nutritionists, geneticists, animal keepers/farmers, veterinarians, etc.) with scientists working along the food chain in the fields of plant and feed science, ecology, and economy contributes to improved problem solving and to developing improved and resilient LF. In summary, the production of more food for more people with fewer resources and emissions is one of the most important challenges for all of those involved in feed and food science and production.

## Figures and Tables

**Table 1:** Intake of milk, meat, and eggs as well as protein of animal origin per capita and year ^1^ and portion (%) of total protein intake (global minimum-values; maximum-values and averages, as well as German values for comparison; kg per capita and year: data base 2005; [15].

Food	Minimum	Average	Maximum
Milk (kg per year)	1.3 (Kongo)	82.1	367.7 (Sweden)
Meat ^2^ (kg per year)	3.1 (Bangladesh)	41.2	142.5 (Luxembourg)
Eggs (kg per year)	0.1 (Kongo)	9.0	20.2 (China)
Edible protein of animal origin (g per capita and day)	1.7 (Burundi)	23.9	69.0 (USA)
Portion of animal protein as % of total protein intake per capita	4.0 (Burundi)	27.9	59.5 (USA)

^1^ Total daily protein intake per person: Burundi 42.5 g, global average 85.7 g, USA 116.0 g; ^2^ Presumptive empty body weight (meat plus bones).

**Table 2:** Crude protein content of some feeds [60] and their human-edible fraction (hef; in %) (taken from Wilkinson [57]) and for three different estimation scenarios from Ert et al. [58].

Feedstuff	Crude Protein (g/kg DM ^3^)	Hef-Fractions (% of CP)	Hef-Fractions (% of CP ^2^)		
			Low	Medium	High
Barley	125	80	40	65	80
Maize	106	80	70	80	90
Wheat	138	80	60	80	100
Soybeans	404	80	50	92	93
Rapeseed meal	406	20	30	59	87
Soybeans meal	513	80	50	71	92
Wheat bran	160	20	0	10	20
Maize silage	86	0	19	29	45
Others ^1^		0	0	0	0

^1^ Other co-products (e.g., sugar beet pulp; brewer’s grains; dried distiller’s grains with solubles, etc.) and roughages (e.g., fresh grass; silages, hay etc.); ^2^ Crude protein; ^3^ Dry matter.

**Table 3:** Protein content of some edible land animal products/food by various authors (in g per kg edible product).

Product/Food Authors	Milk (Cows)	Beef	Pork	Poultry	Eggs
Flachowsky [65]	34	190	150	200	120
GfE ^1^ [66,67,68,69]	34	170–200	157 (129–178)	n.d. ^2^	121 (110–124)
Souci et al. [70]	33.3 (30.8–37.0)	220 ^3^ (206–227)	220 ^3^ (195–240)	199	125
De Vries and de Boer [55]	30	190	190	190	130
Mekonnen and Hoekstra [71]	33	138	105	127	111
Andersen [72]	34	206–212	183–216	182–242	125
Lesschen et al. [73] ^4^	34.4	206	156	206	119
Nijdam et al. [38]	35	200	200	200	130
USDA [74]	34	173	139	186	126

^1^ Gesellschaft für Ernährungsphysiologie; ^2^ no data; ^3^ Muscles only; ^4^ N-content x 6.25.

**Table 4:** Influence of animal species, categories, and performances on yield of edible protein (without considering rearing periods and animal losses) [62,65].

Protein Source (Body Mass)	Performance (per d)	Dry Matter Intake (kg per d)	Forage to Concentrate Ratio (%, DM Basis)	Edible Fraction (% of Product or Body Mass)	Protein in Edible Fraction (g per kg)	Edible Protein Yield (g per d)	Edible Protein Yield (g per kg Body Mass and d)
Dairy cow (650 kg)	2 kg milk 5 kg milk 10 kg milk 20 kg milk 40 kg milk	8 10 12 16 25	100 95/5 90/10 75/25 50/50	95	34	67 163 323 646 1292	0.1 0.25 0.5 1.0 2.0
Dairy goat (60 kg)	0.5 kg milk 1 kg milk 2 kg milk	1 1.5 2	100 90/10 80/20	95	36	17 34 68	0.3 0.55 1.1
Beef cattle (350 kg)	200 g ADG ^1^ 500 g ADG 1000 g ADG 1500 g ADG	6.0 6.5 7.0 7.5	100 95/5 85/15 70/30	50	190	19 48 95 143	0.05 0.14 0.27 0.41
Growing/fattening pig (80 kg)	200 g ADG 500 g ADG 700 g ADG 1000 g ADG	1.5 1.8 2 2.2	30/70 20/80 10/90 0/100	60	150	18 45 63 90	0.22 0.56 0.8 1.1
Chicken for fattening (1.5 kg)	20 g ADG 40 g ADG 60 g ADG	0.06 0.07 0.08	15/85 10/90 0/100	60	200	2.4 4.8 7.2	1.6 3.2 4.8
Laying hen (1.8 kg)	20% LP ^2^ 50% LP 70% LP 90% LP	0.09 0.10 0.11 0.12	30/70 20/80 10/90 0/100	95	120	1.4 3.4 4.8 6.2	0.8 1.9 2.7 3.4

^1^ Average daily gain; ^2^ Laying performance.

**Table 5:** Assumed plant yields for further calculations (kg dry matter per ha and year).

Yield Level	Grassland or Perennial Crops (Roughage)	Arable Land or Cultivated Crops (Concentrate)
A (low)	5000	2000
B (medium)	10,000	5000
C (high)	20,000	10,000

**Table 6:** Model calculations for land use per kg of edible protein depending on animal species/categories, plant yields, and animal performances (for forage to concentrate ratio see Table 4; all concentrates from arable land, no co-products considered).

Protein Source	Animal Yield	Edible Protein Yield (g/d)	Grassland or Perennial Crops (m^2^/kg Protein) ^1^			Arable Land or Cultivated Crops (m^2^/kg Protein)		
			A ^2^	B	C	A	B	C
Cow milk	2 kg per d	67	240	120	60	0	0	0
	5 kg per d	163	120	60	30	15	6	3
	10 kg per d	323	70	35	18	18	8	4
	20 kg per d	646	38	20	9	30	12	6
	40 kg per d	1292	20	10	5	50	20	10
Goat milk	0.5 kg per d	17	120	60	30	0	0	0
	1 kg per d	34	80	40	20	22	9	5
	2 kg per d	68	50	25	13	30	12	6
Beef	200 g ADG ^3^	19	630	315	160	0	0	0
	500 g ADG	48	260	130	65	35	15	7
	1000 g ADG	95	125	60	30	55	22	11
	1500 g ADG	143	75	40	20	80	30	15
Pork	200 g ADG	18	50	25	12	300	120	60
	500 g ADG	45	16	8	4	160	65	32
	700 g ADG	63	8	4	2	140	55	28
	1000 g ADG	90	0	0	0	120	50	24
Chicken meat	20 g ADG	2.4	8	4	2	100	4	20
	40 g ADG	4.8	3	2	1	65	25	13
	60 g ADG	7.2	0	0	0	60	25	12
Eggs	20% LP ^4^	1.4	40	20	10	220	90	45
	50% LP	3.4	12	6	3	110	50	25
	70% LP	4.8	5	2	1	100	40	25
	90% LP	6.2	0	0	0	95	40	20

^1^ Some authors (e.g., [63]) calculated this without perennial crops in non-ruminant (pigs and poultry) feeding; ^2^ Plant yields: Levels A (low), B (medium), and C (high); see Table 5; ^3^ Average daily gain; ^4^ Laying performance.

**Table 7:** Land use per livestock product or protein (in m^2^/kg product and m^2^/kg protein; *n* = 16; [55]).

Food of Animal Origin	Land Use (m^2^/kg Product)	Land Use (m^2^/kg Protein)
Milk	1.1–2.0	33–59
Beef ^1^	27–491	144–258
Pork	8.9–12.1	47–64
Chicken meat	8.1–9.9	42–52
Eggs	4.5–6.2	35–48

^1^ Suckler cows with calves.

**Table 8:** Land use (both total and grassland) per kg product and per kg edible protein [38].

Food of Animal Origin (Number of Studies)	Total Land Use (m^2^/kg Product)	Proportional Grassland Use (m^2^/kg Product)	Total Land Use (m^2^/kg Protein)
Milk (14)	1–2	1	26–54
Beef; allover (26) Industrial systems (11) Suckler herds (8) Extensive pastoral systems (4)	7–420 15–29 33–158 286–420	2–420 2–26 25–140 250–420	37–2100 75–143 164–788 1430–2100
Mutton (5)	20–33	18–30	100–165
Pork (11)	8–15	Not applicable	40–75
Chicken meat (5)	5–8	Not applicable	23–40
Eggs (5)	4–7	Not applicable	29–52

**Table 9:** Model calculations for the land use per kg edible protein depending on animal species/categories, plant yields, animal performances, and co-products from the agriculture, food, and fuel industries (see Table 3, Table 4 and Table 5 for further details).

Protein Source	Animal Yield	Edible Protein Yield (g/Day)	Grassland or Perennial Crops (m^2^/kg Protein)			Arable Land or Cultivated Crops (m^2^/kg Protein)		
			Plant Yield Level B ^1^					
			Replacement by Co-Products (%)					
			10	20	30	15	20	45
Cow milk	2 kg per d	67	108	96	84	0	0	0
	5 kg per d	163	54	48	42	5	4	3
	10 kg per d	323	32	28	24	7	6	5
	20 kg per d	646	16	14	12	11	10	8
	40 kg per d	1292	9	8	7	17	14	11
Goat milk	0.5 kg per d	17	54	48	42	0	0	0
	1 kg per d	34	34	28	21	8	6	5
	2 kg per d	68	20	18	14	10	8	6
Beef	200 g ADG ^2^	19	280	250	220	0	0	0
	500 g ADG	48	115	105	90	13	10	8
	1000 g ADG	95	54	48	42	19	15	12
	1500 g ADG	143	36	32	28	26	21	16
Pork	200 g ADG	18	22	20	18	102	84	65
	500 g ADG	45	7	6	5	55	46	36
	700 g ADG	63	4	3	3	47	39	30
	1000 g ADG	90	0	0	0	42	35	28
Chicken meat	20 g ADG	2.4	4	3	3	34	28	22
	40 g ADG	4.8	2	2	1	21	18	14
	60 g ADG	7.2	0	0	0	21	18	14
Eggs	20% LP ^3^	1.4	18	16	14	76	63	50
	50% LP	3.4	5	5	4	42	35	28
	70% LP	4.8	2	2	1	34	28	22
	90% LP	6.2	0	0	0	34	28	22

^1^ Plant yields: Levels A (low), B (medium), and C (high); see Table 3 and Table 5; ^2^ Average daily gain; ^3^ Laying performance.

**Table 10:** Calculation to the net protein contribution of milk production to the human food chain under consideration of various amounts of co-products in concentrate (on the basis of the data in Table 2 and Table 4).

Milk Yield	Total DM-Intake	Concentrate Intake	Co-Products in Concentrate	Human Edible Protein Input	Human Edible Protein Output	Proportion Output to Input
(kg per d)	(kg/d)	(kg DM/d)	(%)	(g/d)	(g/d)	(g/g)
2	8	0	0	0	67	-
5	10	0.5	100 ^1^	8	163	20
10	12	1.2	100 ^1^	96	323	3.4
20	16	4.0	50 ^2^	262	646	2.4
40	25	12.5	25 ^3^	1450	1292	0.9

^1^ 50% concentrate wheat bran; 50% dried sugar beet pulp; ^2^ 25% concentrate wheat bran; 25% dried sugar beet pulp; 30% concentrate as cereals; 10% soybean meal; 10% rapeseed meal; ^3^ 12.5% concentrate wheat bran; 12.5% dried sugar beet pulp; 50% concentrate as cereals; 15% soybean meal; 10% rapeseed meal (see Table 2; hef-fractions by [57]).

**Table 11:** Calculation of the net protein contribution of food of animal origin to the human food chain without co-products and under consideration of 50% of concentrate based on co-products (based on data of Table 2 and Table 4).

Protein for Human Alimentation	Animal Yield	DM Intake	Concentrate Intake ^1^	Co-Products in Concentrate ^2^	Human Edible Protein Input ^3^ Output ^4^		Proportion Output to Input
	(per d)	(kg/d)	(kg DM/d)	(kg DM/d)	(g/d)	(g/d)	(g/g)
Cow Milk	20 kg	16	4	0 2	493 262	646	1.3 2.4
Beef	1000 g ADG ^5^	7	1.05	0 0.52	130 70	95	0.7 1.3
Pork	700 g ADG	2	1.8	0 0.9	224 127	63	0.3 0.5
Chicken	60 g ADG	0.08	0.08	0 0.04	10 6	7.2	0.7 1.2
Eggs	70% LP ^6^	0.11	0.1	0 0.05	12 7	4.8	0.4 0.7

^1^ 80% cereals; 20% protein sources (soybeans, rapeseed); ^2^ 50% co products; 30% cereals; 20% protein sources (soybeans, rapeseed); ^3^ see Table 2; (hef-fractions by [57]); ^4^ see Table 4; ^5^ Average daily gain; ^6^ Laying performance.

## References

[1] Food and Agriculture Organization of the United Nations (FAO), International Fund for Agricultural Development (IFAD), World Food Programme (WFP) (2015). The State of Food Insecurity in the World 2015. Meeting the 2015 International Hunger Targets: Taking Stock of Uneven Progress.

[2] Fedoroff N.V., Battisti D.S., Beachy R.N., Cooper P.J., Fischhoff D.A., Hodges C.N., Knauf V.C., Lobell D., Mazur B.J., Molden D. (2010). Radically rethinking agriculture for the 21st century. Science.

[3] Godfray H.C., Beddington J.R., Crute I.R., Haddad L., Lawrence D., Muir J.F., Pretty J., Robinson S., Thomas S.M., Toulmin C. (2010). Food security: The challenge of feeding 9 billion people. Science.

[4] Foley J.A., Ramankutty N., Brauman K.A., Cassidy E.S., Gerber J.S., Johnston M., Mueller N.D., O’Connell C., Ray D.K., West P.C. (2011). Solutions for a cultivated planet. Nature.

[5] Aiking H. (2014). Protein production: Planet, profit, plus people?. Am. J. Clin. Nutr..

[6] Beddington J.R., Asaduzzaman M., Clark M., Fernández A., Guillou M., Jahn M., Erda L., Mamo T., van Bo N., Nobre C.A. (2012). Achieving Food Security in the Face of Climate Change: Final Report from the Commission on Sustainable Agriculture and Climate Change.

[7] Schlink A.C., Nguyen M.L., Viljoen G.J. (2010). Water requirements for livestock production: A global perspective. Rev. Sci. Tech..

[8] Deikman J., Petracek M., Heard J.E. (2012). Drought tolerance through biotechnology: Improving translation from the laboratory to farmers’ fields. Curr. Opin. Biotechnol..

[9] Hoekstra A.Y., Chapagain A.K., Zhang G.P. (2016). Water footprints and sustainable water allocation. Sustainability.

[10] Hall D.C., Hall J.V. (1984). Concepts and measures of natural-resource scarcity with a summary of recent trends. J. Environ. Econ. Manag..

[11] Scholz R.W., Wellmer F.W. (2013). Approaching a dynamic view on the availability of mineral resources: What we may learn from the case of phosphorus?. Glob. Environ. Chang..

[12] Aerts S., Potthast T., Meisch S. (2012). Agriculture’s 6 F’s and the need for more intensive agriculture. Climate Change and Sustainable Development.

[13] Boonen R., Aerts S., de Tavernier L., Potthast T., Meisch S. (2012). Which sustainability suits you?. Climate Change and Sustainable Development.

[14] Food and Agriculture Organization of the United Nations (FAO) (2009). How to Feed the World in 2050.

[15] Food and Agriculture Organization of the United Nations (FAO) (2009). The State of Food and Agriculture. Livestock in the Balance. State of Foods and Agriculture.

[16] Alexandratos N., Bruinsma J. (2012). World Agriculture towards 2030/2050: The 2012 Revision.

[17] Kastner T., Rivas M.J., Koch W., Nonhebel S. (2012). Global changes in diets and the consequences for land requirements for food. Proc. Natl. Acad. Sci. USA.

[18] Cassidy E.S., West P.C., Gerber J.S., Foley J.A. (2013). Redefining agricultural yields: From tonnes to people nourished per hectare. Environ. Res. Lett..

[19] Kebreab E. (2013). Sustainable Animal Agriculture.

[20] Smil V. (2001). Feeding the World: A Challenge for the Twenty-First Century.

[21] Ertl P., Zebeli Q., Zollitsch W., Knaus W. (2015). Feeding of by-products completely replaced cereals and pulses in dairy cows and enhanced edible feed conversion ratio. J. Dairy Sci..

[22] World Health Organization (WHO), Food and Agriculture Organization of the United Nations (FAO), United Nations University (UNU) (2002). Protein and Amino Acid Requirements in Human Nutrition.

[23] D’Mello J.P.F. (2012). Amino Acids in Human Nutrition and Health.

[24] FAO (Food and Agriculture Organization of the United Nations) (2010). Greenhouse Gas Emissions from the Dairy Sector.

[25] Wennemer H., Flachowsky G., Hoffmann V. (2006). Protein, Population, Politics—How Protein Can Be Supplied Sustainable in the 21st Century.

[26] Reynolds L.P., Wulster-Radcliffe M.C., Aaron D.K., Davis T.A. (2015). Importance of animals in agricultural sustainability and food security. J. Nutr..

[27] Waterlow J.C. (1999). The mysteries of nitrogen balance. Nutr. Res. Rev..

[28] Jackson A.A., Truswell S., Mann J., Truswell S. (2007). Protein. Essentials of Human Nutrition.

[29] Rand W.M., Pellett P.L., Young V.R. (2003). Meta-analysis of nitrogen balance studies for estimating protein requirements in healthy adults. Am. J. Clin. Nutr..

[30] DGE (Deutsche Gesellschaft für Ernährung), ÖGE (Österreichische Gesellschaft für Ernährung), SGE (Schweizerische Vereinigung für Ernährung) (2000). Referenzwerte für die Nährstoffzufuhr.

[31] Avadi A., Freon P. (2013). Life cycle assessment of fisheries: A review for fisheries scientists and managers. Fish. Res..

[32] Keyzer M.A., Merbis M.D., Pavel I.F.P.W., van Wesenbeeck C.F.A. (2005). Diet shifts towards meat and the effects on cereal use: Can we feed the animals in 2030?. Ecol. Econ..

[33] Guyomard H., Manceron S., Peyraud J.L. (2013). Trade in feed grains, animals, and animal products: Current trends, future prospects, and main issues. Anim. Front..

[34] Wu G., Fanzo J., Miller D.D., Pingali P., Post M., Steiner J.L., Thalacker-Mercer A.E. (2014). Production and supply of high-quality food protein for human consumption: Sustainability, challenges, and innovations. Ann. N. Y. Acad. Sci..

[35] Guyomard H., Darcy-Vrillon B., Esnouf C., Marin M., Russel M., Guillou M. (2012). Eating patterns and food systems: Critical knowledge requirements for policy design and implementation. Agric. Food Secur..

[36] Pimentel D., Pimentel M. (2003). Sustainability of meat-based and plant-based diets and the environment. Am. J. Clin. Nutr..

[37] Baroni L., Cenci L., Tettamanti M., Berati M. (2007). Evaluating the environmental impact of various dietary patterns combined with different food production systems. Eur. J. Clin. Nutr..

[38] Nijdam D., Rood T., Westhoek H. (2012). The price of protein: Review of land use and carbon footprints from life cycle assessments of animal food products and their substitutes. Food Policy.

[39] Peters C.J., Wilkins J.L., Fick G.W. (2007). Testing a complete-diet model for estimating the land resource requirements of food consumption and agricultural carrying capacity: The new york state example. Renew. Agric. Food Syst..

[40] Westhoek H., Lesschen J.P., Rood T., Wagner S., de Marco A., Murphy-Bokern D., Leip A., van Grinsven H., Sutton M.A., Oenema O. (2014). Food choices, health and environment: Effects of cutting Europe’s meat and dairy intake. Glob. Environ. Chang..

[41] Intergovernmental Panel on Climate Change (IPCC) (2006). Guidelines for National Greenhouse Gas Inventories; Agriculture, Forestry and Other Land Use.

[42] Williams A.G., Audsley E., Sanders D.L. (2006). Determining the Environmental Burdens and Resource Use in the Production of Agricultural and Horticultural Commodities.

[43] Hristov A.N., Oh J., Lee C., Meinen R., Montes F., Ott T., Firkins J., Rotz A., Dell C., Adesogan A.T. (2013). Mitigation of Greenhouse Gas Emissions in Livestock Production—A Review of Technical Options for Non-CO_2_ Emissions.

[44] Flachowsky G., Malik P.K., Malik P.K., Bhatta R., Takahashi J., Kohn R.A., Prasad C.S. (2015). Carbon footprints for food of animal origin. Livestock Production and Climate Change.

[45] Gerber P.J., Steinfeld H., Henderson B., Mollet A., Opio C., Dijkman F., Falcucci A., Tempio G. (2013). Tackling Climate Change through Livestock—A Global Assessment of Emissions and Mitigation Opportunities.

[46] Friel S., Dangour A.D., Garnett T., Lock K., Chalabi Z., Roberts I., Butler A., Butler C.D., Waage J., McMichael A.J. (2009). Public health benefits of strategies to reduce greenhouse-gas emissions: Food and agriculture. Lancet.

[47] Wheeler T., Reynolds C. (2013). Predicting the risks from climate change to forage and crop production for animal feed. Anim. Front..

[48] Niemann H., Kuhla B., Flachowsky G. (2011). Perspectives for feed-efficient animal production. J. Anim. Sci..

[49] Makkar H.P.S., Tran G., Heuze V., Ankers P. (2014). State-of-the-art on use of insects as animal feed. Anim. Feed Sci. Technol..

[50] Makkar H.P.S., Ankers P. (2014). Towards sustainable animal diets: A survey-based study. Anim. Feed Sci. Technol..

[51] Bruinsma J. (2009). The resource outlook to 2050: By how much do land, water and crop yields need to increase by 2050?. Expert Meeting on How to Feed the World in 2050.

[52] Food and Agriculture Organization of the United Nations (FAO) (2013). Dietary Protein Quality Evaluation in Human Nutrition.

[53] Bessou C., Ferchaud F., Gabrielle B., Mary B. (2011). Biofuels, greenhouse gases and climate change. A review. Agron. Sustain. Dev..

[54] Elferink E.V., Nonhebel S. (2007). Variations in land requirements for meat production. J. Clean. Prod..

[55] De Vries M., de Boer I.J.M. (2010). Comparing environmental impacts for livestock products: A review of life cycle assessments. Livest. Sci..

[56] Zollitsch W., Hörtenhuber S., Piringer G. (2012). Life cycle assessment—Aussagekraft und grenzen im kontext tierischer produktionssysteme. Verband Deutscher Landwirtschaftlicher Untersuchungs- und Forschungsanstalten.

[57] Wilkinson J.M. (2011). Re-defining efficiency of feed use by livestock. Animal.

[58] Ertl P., Klocker H., Hortenhuber S., Knaus W., Zollitsch W. (2015). The net contribution of dairy production to human food supply: The case of austrian dairy farms. Agric. Syst..

[59] Van Zanten H.H.E., Mollenhorst H., Klootwijk C.W., van Middelaar C.E., de Boer I.J.M. (2016). Global food supply: Land use efficiency of livestock systems. Int. J. Life Cycle Assess..

[60] Jeroch H., Flachowsky G., Weissbach F. (1993). Futtermittelkunde.

[61] Food and Agriculture Organization of the United Nations (FAO), World Health Organization (WHO) (1991). Protein Quality Evaluation: Report of the Joint Fao/Who Expert Consultation, Bethesda, MD, USA, 4–8 December 1989.

[62] Flachowsky G., Kamphues J. (2012). Carbon footprints for food of animal origin: What are the most preferable criteria to measure animal yields?. Animals.

[63] Peters G.M., Rowley H.V., Wiedemann S., Tucker R., Short M.D., Schulz M. (2010). Red meat production in australia: Life cycle assessment and comparison with overseas studies. Environ. Sci. Technol..

[64] Doreau M., van der Werf H.M.G., Micol D., Dubroeucq H., Agabriel J., Rochette Y., Martin C. (2011). Enteric methane production and greenhouse gases balance of diets differing in concentrate in the fattening phase of a beef production system. J. Anim. Sci..

[65] Flachowsky G. (2002). Efficiency of energy and nutrient use in the production of edible protein of animal origin. J. Appl. Anim. Res..

[66] GfE (Gesellschaft für Ernährungsphysiologie) (1995). Recommendations for Energy and Nutrient Requirements of Beef Cattle.

[67] GfE (Gesellschaft für Ernährungsphysiologie) (1999). Recommendations for Energy and Nutrient Requirements of Laying Hens and Broilers.

[68] GfE (Gesellschaft für Ernährungsphysiologie) (2001). Recommendations for Energy and Nutrient Requirements of dairy Cattle and Heifers.

[69] GfE (Gesellschaft für Ernährungsphysiologie) (2008). Recommendations for the Supply of Energy and Nutrients to Pigs.

[70] Souci S.W., Fachmann W., Kraut H. (2016). Food Composition and Nutrition Tables: Die Zusammensetzung der Lebensmittel, Nährwert-Tabellen la Composition des Aliments Tableaux des Valeurs Nutritives.

[71] Mekonnen M.M., Hoekstra A.V. (2010). The Green, Blue and Grey Water Footprint of Farm Animals and Animal Products.

[72] Andersen G. (2011). Food Table for the Practice: The Little Souci-Fachmann-Kraut.

[73] Lesschen J.P., van den Berg M., Westhoek H.J., Witzke H.P., Oenema O. (2011). Greenhouse gas emission profiles of european livestock sectors. Anim. Feed Sci. Technol..

[74] USDA (United States Department of Agriculture) Agricultural Research Service; National Nutrient Database for Standard Reference. https://ndb.nal.usda.gov/ndb/.

[75] Mekonnen M.M., Hoekstra A.Y. (2012). A global assessment of the water footprint of farm animal products. Ecosystems.

[76] NRC (National Research Counsil) (2001). Nutrient Requirements of Dairy Cattle: Seventh Revised Edition, 2001.

[77] GfE (Gesellschaft für Ernährungsphysiologie) (2014). Recommendations for the Supply of Energy and Nutrients to Horses.

[78] Flachowsky G. (2013). Animal Nutrition with Transgenic Plants.

[79] Van Soest P.J. (1994). Nutritional Ecology of the Ruminant.

[80] Becker M., Nehring K. (1969). Handbook of Feeds. Handbuch der Futtermittel.

[81] Beyer M., Jentsch W., Chudy A. (2003). Rostock Feed Evaluation System: Reference Numbers of Feed Value and Requirement on the Base of Net Energy.

[82] Wheeler J.L., Wheeler J.L., Pearson C.J., Roberts G.E. (1987). Pastures and pasture research in Southern Australia. Temperate Pastures: Their Production, Use and Management.

[83] Gill M., Makkar H.P.S., Beever D. (2013). Converting Feed into Human Food: The Multiple Dimensions of Efficiency; In Optimization of Feed Use Efficiency in Ruminant Production Systems. Proceedings of the FAO Symposium, 27 November 2012, Bangkok, Thailand.

[84] Taube F., Gierus M., Hermann A., Loges R., Schönbach P. (2014). Grassland and globalization-challenges for north-west European grass and forage research. Grass Forage Sci..

[85] Ertl P., Zebeli Q., Zollitsch W., Knaus W. (2016). Feeding of wheat bran and sugar beet pulp as sole supplements in high-forage diets emphasizes the potential of dairy cattle for human food supply. J. Dairy Sci..

[86] Kratli S., Huelsebusch C., Brooks S., Kaufmann B. (2013). Pastoralism: A critical asset for food security under global climate change. Anim. Front..

[87] Powell J.M., MacLeod M., Vellinga T.V., Opio C., Falcucci A., Tempio G., Steinfeld H., Gerber P. (2013). Feed-milk-manure nitrogen relationships in global dairy production systems. Livest. Sci..

[88] Gill M., Smith P., Wilkinson J.M. (2010). Mitigating climate change: The role of domestic livestock. Animal.

[89] Opio C., Gerber P., Mottet A., Falcucci A., Tempio G., MacLeod M., Vellinga T., Henderson B., Steinfeld H. (2013). Greenhouse Gas Emissions from Ruminant Supply Chains—A Global Life Cycle Assessment.

[90] Soussana J.F., Loiseau P., Vuichard N., Ceschia E., Balesdent J., Chevallier T., Arrouays D. (2004). Carbon cycling and sequestration opportunities in temperate grasslands. Soil Use Manag..

[91] Soussana J.F., Tallec T., Blanfort V. (2010). Mitigating the greenhouse gas balance of ruminant production systems through carbon sequestration in grasslands. Animal.

[92] Sundstol F., Owen E. (1984). Straw and Other Fibrous by Products as Feed.

[93] Flachowsky G. (1987). Stroh als Futtermittel: Ergebnisse und Erfahrungen Bei der Strohaufbereitung und Beim Einsatz von Unterschiedlich Behandeltem Stroh als Futtermittel.

[94] Kling M., Wöhlbier W. (1983). Trade Feestuffs (in German: Handelsfuttermittel).

[95] Food and Agriculture Organization of the United Nations (FAO) (2012). Biofuel Co-Products as Livestock Feed—Opportunities and Challenges.

[96] Gerber P., Vellinga T., Opio C., Steinfeld H. (2011). Productivity gains and greenhouse gas emissions intensity in dairy systems. Livest. Sci..

[97] Windisch W., Fahn C., Brugger D., Deml M., Buffler M. (2013). Strategies for sustainable animal nutrition. Züchtungskunde.

[98] Reynolds C.K., Crompton L.A., Mills J.A.N. (2011). Improving the efficiency of energy utilisation in cattle. Anim. Prod. Sci..

[99] Bradford G.E. (1999). Contributions of animal agriculture to meeting global human food demand. Livest. Prod. Sci..

[100] Nguyen T.L.T., Hermansen J.E., Mogensen L. (2010). Environmental consequences of different beef production systems in the eu. J. Clean. Prod..

[101] Bockisch F.-J., Röver M. (2000). Bewertung von Verfahren der Ökologischen und Konventionellen Landwirtschaftlichen Produktion im Hinblick auf den Energieeinsatz und Bestimmte Schadgasemissionen: Studie als Sondergutachten im Auftrag des Bundesministeriums für Ernährung, Landwirtschaft und Forsten.

[102] Ertl P., Knaus W., Zollitsch W. (2016). An approach to including protein quality when assessing the net contribution of livestock to human food supply. Animal.

[103] NRC (National Research Council of the National Academies) (2015). Critical Role of Animal Science Research in Food Security and Sustainability.

[104] EFSA (European Food Safety Authority) (2015). Risk profile related to production and consumption of insects as food and feed. EFSA J..

[105] Food and Agriculture Organization of the United Nations (FAO) (2013). Food Wastage Footprint—Impacts on Natural Resources.

[106] Blanke M. (2015). Challenges of reducing fresh produce waste in Europe—From farm to fork. Agriculture.

